# Purely Endoscopic Resection of Cavernoma of the Septum Pellucidum

**DOI:** 10.1055/s-0039-1693082

**Published:** 2019-06-17

**Authors:** Sara Baldo, Salima Magrini, Leonello Tacconi

**Affiliations:** 1 Department of Neurosurgery, University Hospital of Trieste, Trieste, Italy

**Keywords:** endoscopy, cavernoma, intraventricular

## Abstract

The intraventricular location of a cavernoma is a rare entity and accounts for approximately 2.5% of all cavernomas of the central nervous system. They are commonly found in the lateral ventricle followed by the third and fourth ventricles. The location in the septum pellucidum is rare, and only four cases have been reported in the international literature. An open craniotomy was performed in all these cases. To the best of our knowledge, this is the first case of a cavernoma of the septum pellucidum successfully resected using a purely endoscopic transventricular approach.

Cavernomas (CVs) are common cerebral lesions, most frequently located in the supratentorial (74–90%) rather than the infratentorial (10–26%) region. 1 2 They can be single as well as multiple lesions and can present themselves sporadically or can be an expression of a genetic disease: three genes have been identified for the inherited form. 3 4 The intraventricular location is rare and accounts for approximately 2.5% of all CVs of the central nervous system, 5 with the atrium being the most common site. 6 A CV arising from the septum pellucidum is a rare entity, and only four cases have been reported in the international literature. 7 8 9 10 Generally speaking, CVs may be detected incidentally or present with seizures, headaches, or focal neurologic deficits due to hemorrhage. The clinical features largely depend on the location of the CV. In the supratentorial compartment, seizures are the most common symptoms in 39 to 79% of the patients. 11 The intraventricular location may, most commonly, present either with hemorrhage (14–22%), seizures (14%), or hydrocephalus, or may even be asymptomatic. 4 10 12 CVs of the septum pellucidum do not have any specific clinical features. The standard treatment for CVs is surgical removal, although, most recently, even stereotactic radiotherapy has been used. 3 The transcranial transcortical or the transcranial interhemispheric approaches are the surgical classical routes used for CVs located within the septum pellucidum. A minimally invasive surgical technique, such as the endoscopic one, can be used to decrease the surgical brain manipulation as well.

## Case Report

### History

**Fig. 1:**
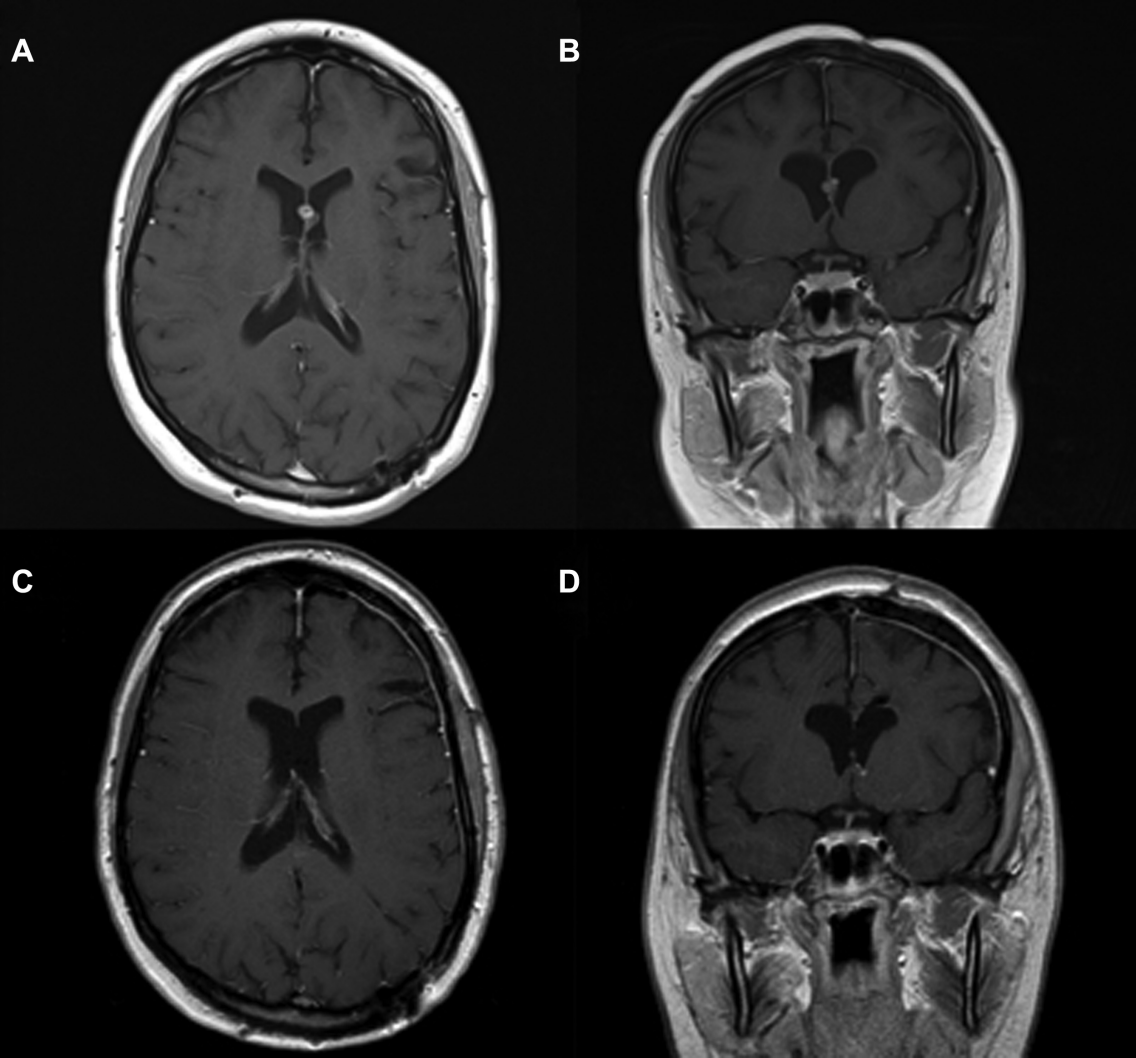
( **A** ) Axial and ( **B** ) coronal T1-weighted preoperative magnetic resonance (MR) images reveal an 18-mm multilobulated mass with heterogeneous signal intensity located at the septum pellucidum. Postoperative ( **C** ) axial and ( **D** ) coronal T1-weighted image shows no cavernoma remnant.

The patient's clinical history started when she was 13 years old and presented with a generalized tonic–clonic seizure, which lead to a magnetic resonance imaging (MRI) of the brain with the subsequent diagnosis of multiple intracerebral CVs: a left frontal intraparenchymal one (35 mm in diameter) and a left posterior temporal one, both within the parenchyma (23 mm in diameter), and an intraventricular one (30 mm in diameter). Despite the best medical treatment, the epilepsy was not well controlled and the patient had up to three to four epileptic attacks per week. This case was discussed several times at our multidisciplinary meeting, as well as with the patients and the parents. The final decision was to remove the largest and the apparently symptomatic CV, and this decision was guided by a video-EEG (electroencephalogram). The left frontal CV was removed at the age of 14 years, with epilepsy symptoms being temporarily improved. Unfortunately, after 10 months, she started to complain of epilepsy again, with a clinical absence type behavior, pointed for temporal lobe origin type of seizures. Thus, a few months later, the left posterior temporal lesion was removed as well. The second operation gave very good medical results in terms of seizures control. The episodes dropped to one or two focal seizures per year. The third lesion, the intraventricular one, was followed up with a yearly MRI scan. At the age of 21, because the lesion had increased in size (∼8 mm) and because of the patient's desire, we decide to remove it using a transcranial interhemispheric approach. The operation was uncomplicated, and the patient was discharged home a week after the procedure. At that stage, no other lesions were present, and in the following 10 years, the follow-up MRI scans did not show any recurrence or new CVs. When she was 32 years old, on the yearly follow-up scan, a newly developed lesion was identified. This lesion, suspicious for CV, was small (6 mm) and located within the septum pellucidum. Because of its small size, the location, and the absence of symptoms, a conservative treatment option was followed. Unfortunately, the lesion doubled in size in the following 18 months and therefore the patient was very adamant about having it removed ( Fig. 1 ). We were a bit reluctant because the patient was completely asymptomatic and had not had any epileptic attack for 10 years. Upon neurologic examination, she presented no issues. Finally, we took the decision to remove the lesion and we started to discuss how to approach it. We were wondering whether to use the same interhemispheric approach with the possibility of encountering scar tissue or if it was better to use a new surgical route such as a transcortical one. Finally, we decided to use something completely different and we opted for a transcortical endoscopic approach.

### Operation

**Fig. 2:**
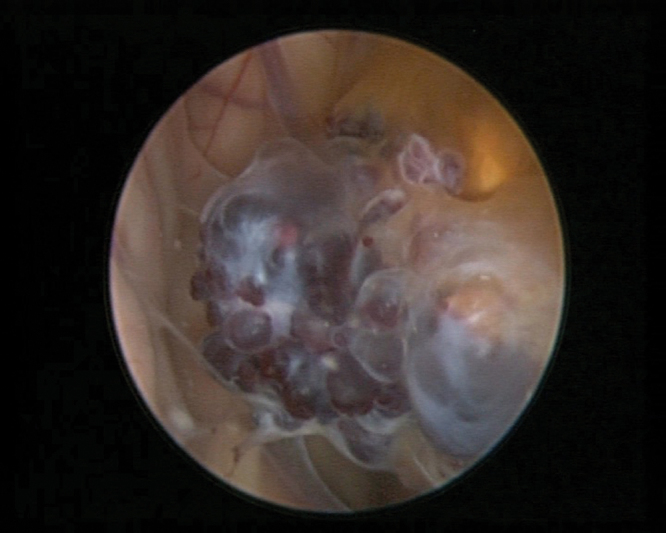
Intraoperative photograph showing a neuroendoscopic view of a cavernoma.

With the patient in the supine position through a single burr hole, placed slightly more laterally in relation to Kocher point, a purely endoscopic approach was performed and the lesion was completely removed ( Video 1 ). A rigid endoscope was used and guided by the neuronavigation. Upon inspection, the lesion ( Fig. 2 ) presented with two veins attached to it (one rostral and the other caudal). The removal began with the coagulation and dissection of the septum pellucidum superior to the CV location. After accurate coagulation and section of the caudal vein, using endoscopic forceps allowed the creation of a “pedunculated” CV. The insertion of an endoscopic rongeur in the space between the peel-away cannula and the endoscope allowed keeping the CV in place, avoiding its fluctuation in the ventricles. This maneuver allowed the exposition and easy dissection of the rostral vein, which, eventually, was coagulated and cut. The CV was then freed from the surrounding tissue and finally removed. An external ventricular drainage was precautionary left in the right ventricle just for 24 hours.

**Video 1**

Surgical video of a purely endoscopic resection of a cavernoma.

### Pathological Findings

The histological examination revealed multiple dilated and congested vascular spaces lined by the endothelium, confirming the diagnosis of a CV.

### Postoperative Course

The postoperative course was uneventful, and the patient was discharged home 2 days later. Serial follow-up MRI scans did not show any new or recurrent lesion at 5 years follow-up.

## Discussion

The management of intracerebral CVs may be complex and is related to the specific location and symptoms. From a general point of view, surgery is indicated in patients with intractable seizures, recurrence of hemorrhages, focal deficits, the presence of mass effect, and lesions increasing in size. Patients with asymptomatic lesions located in eloquent areas may be observed. 10 Intraventricular lesions may present a technical problem further on. The standard approaches used for lesions in this location (lateral and III ventricles) are the transcranial transcortical approach, which is technically easier but requires large ventricles and a corticotomy to enter the brain, which may lead to epilepsy. The other approach used is the transcranial interhemispheric transcallosal route. This approach is more anatomical and does not require large size ventricles, but it is technically more demanding. In addition, if the callosotomy is too posterior or too extensive, it can give way to a disconnection syndrome problem. An alternative way, a less invasive approach, may be the use of an endoscope as a helping tool to the microscope or purely endoscopic approach. The endoscopy technique is being increasingly used for the treatment of intraventricular tumors, but its use in the excision of an intraventricular CV still generates fear and reluctance among neurosurgeons due to the vascular nature of these lesions and the possible difficulty in achieving a good hemostasis. 13 14 15 Microsurgical excision is thus considered the standard treatment and a safer technique compared with neuroendoscopy. 14 16 To the best of our knowledge, this is the first case of a septum pellucidum CV resected purely endoscopically. In this case, the presence of three previous different craniotomies (among these also a transcallosal approach) made the endoscopic resection technique to go through a new surgical corridor, avoiding the presence of the inevitable scar tissue and keeping the surgical act to a minimum. Our choice was also helped by the small size of the CV. However, we were prepared to convert our approach into an open procedure in case of technical problems or bleeding that could obscure our vision. From this case, instead, we have learned that the use of the endoscope may allow for a better visualization all around the lesion if compared with the microscope. As for many clinical situations, the most important criterion is patient selection as well as the surgeon's experience. The use of minimally invasive surgery permits a better surgical outcome in terms of prompt recovery and reduced morbidity.

We believe that this approach should be kept in mind, especially for small lesions located in deep anatomical regions, which would require, otherwise, a more demanding microsurgical open exposure.
